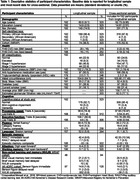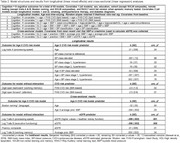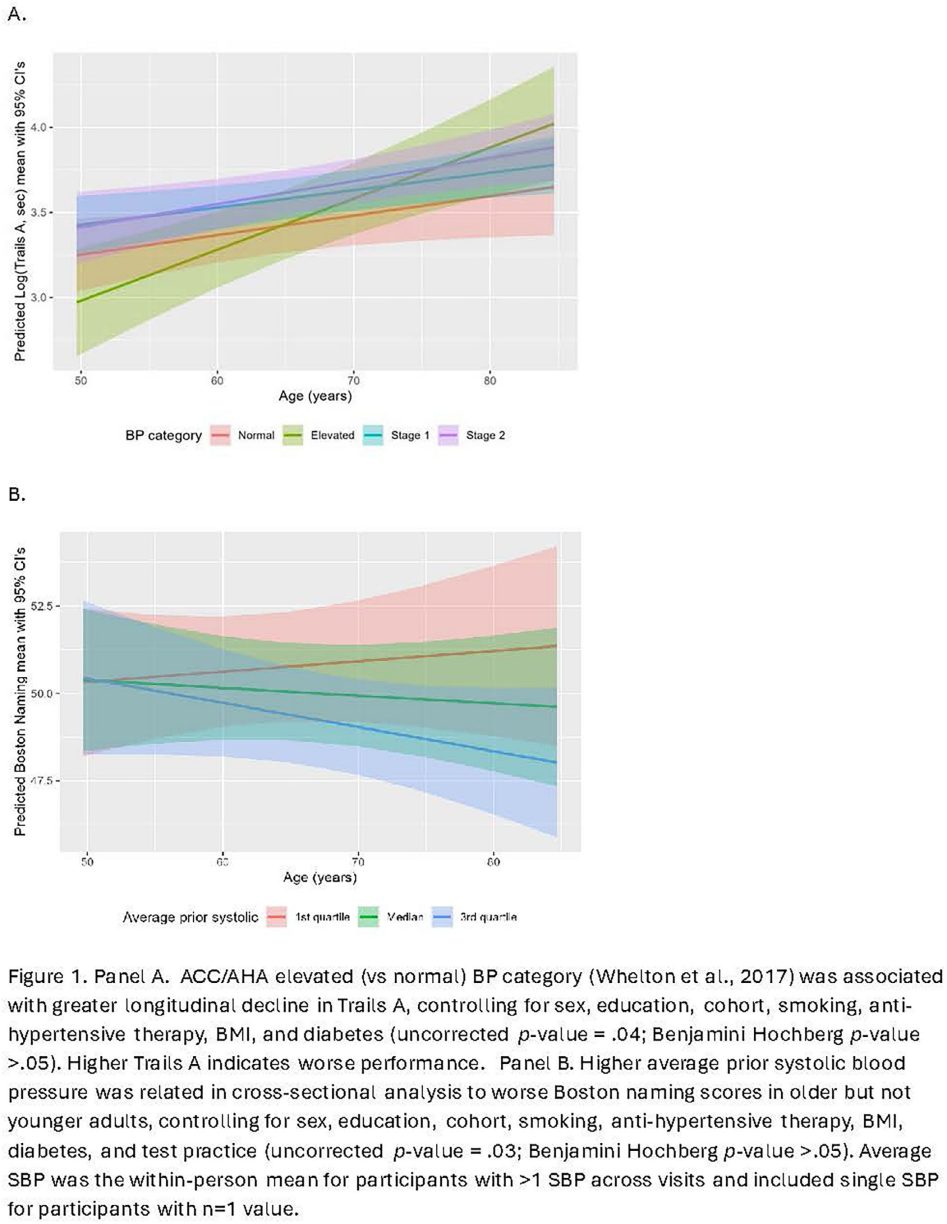# The association of cardiovascular disease risk and renal function to cognition in middle‐aged to older adult Black Americans: Findings from the African Americans Fighting Alzheimer’s in Midlife (AA‐FAIM) study

**DOI:** 10.1002/alz.093448

**Published:** 2025-01-09

**Authors:** Gilda E. Ennis, Derek L. Norton, Hector Salazar, Fabu P. Carter, Erin M. Jonaitis, Rebecca E. Langhough, Nathaniel A. Chin, Megan L. Zuelsdorff, Sanjay Asthana, Sterling C. Johnson, Carey E. Gleason

**Affiliations:** ^1^ Wisconsin Alzheimer’s Disease Research Center, University of Wisconsin School of Medicine and Public Health, Madison, WI USA; ^2^ Wisconsin Alzheimer’s Disease Research Center, Madison, WI USA; ^3^ University of Wisconsin School of Nursing, Madison, WI USA; ^4^ Division of Geriatrics, Department of Medicine, University of Wisconsin School of Medicine and Public Health, Madison, WI USA; ^5^ Wisconsin Alzheimer’s Institute, University of Wisconsin School of Medicine and Public Health, Madison, WI USA; ^6^ Wisconsin Alzheimer's Disease Research Center, University of Wisconsin School of Medicine and Public Health, Madison, WI USA

## Abstract

**Background:**

Non‐Hispanic Black Americans (BA) have increased prevalence of cardiovascular disease (CVD) risk factors and elevated risk for end‐stage renal disease (ESRD). CVD risk factors, and potentially ESRD, heighten dementia risk; however, the association of CVD risk and kidney function to cognition in cognitively unimpaired (CU) BA adults remains understudied. We tested whether global CVD risk, individual CVD risk factors, and less healthy kidney function moderated associations between age and cognitive performance in middle‐aged to older adult BAs who were CU at baseline.

**Method:**

BAs were enrolled in AA‐FAIM, an ancillary study of the Wisconsin Alzheimer’s Disease Research Center (WI‐ADRC) and Wisconsin Registry for Alzheimer’s Prevention (WRAP) (longitudinal N = 192; cross‐sectional N = 323; Table 1). Exclusion criteria included baseline MCI/dementia, stroke, heart attack, or heart failure history. Baseline CVD risk measures were tested as moderators of age‐related cognitive change in mixed models: Framingham Heart Study (FHS) 10‐year CVD risk, systolic blood pressure (SBP), American College of Cardiology/American Heart Association BP classification (normal, elevated, stage 1 and 2 hypertension), waist circumference, and triglyceride/HDL ratio. Cognitive outcomes included processing speed, fluency, executive function, language, episodic memory, attention, working memory, and non‐verbal learning/memory. Regression tested whether average prior SBP and estimated glomerular filtration rate (eGFR) moderated the association between age and cognition. See Table 2 for models. Benjamini Hochberg (BH) correction applied.

**Result:**

24.1% had high CVD risk (FHS CVD > .20). Results suggested a relationship between elevated (vs. normal) BP and greater aging‐related decline in processing speed, and in older adults, an association between higher average prior SBP and lower language scores (Figure 1). Tested without age x risk interaction, results suggested relationships between FHS CVD risk and worse attention and working memory and relationships between lower eGFR and worse fluency, processing speed, and working memory. Latter two eGFR results survived comparisons’ correction but all others did not (Table 2).

**Conclusion:**

Results suggest elevated BP and CVD risk and less healthy kidney function had deleterious effects on cognition in the AA‐FAIM sample; however, most findings did not survive comparisons’ correction, perhaps due to insufficient power. The impact of kidney function on neural structure requires further investigation.